# The design of X-band EPR cavity with narrow detection aperture for in vivo fingernail dosimetry after accidental exposure to ionizing radiation

**DOI:** 10.1038/s41598-021-82462-3

**Published:** 2021-02-08

**Authors:** Junwang Guo, Xiaoxiao Luan, Ye Tian, Lei Ma, Xiaoguang Bi, Jierui Zou, Guofu Dong, Ye Liu, Yonggang Li, Jing Ning, Ke Wu

**Affiliations:** 1 Beijing Institute of Radiation Medicine, Beijing Key Laboratory of Radiation Biology (No. BZ0325), Beijing, 100850 China; 2 Department of Biomedical Engineering, Peking University Third Hospital, Beijing, 100083 China

**Keywords:** Biophysical methods, Assay systems, Biomedical engineering, Techniques and instrumentation

## Abstract

For the purpose of assessing the radiation dose of the victims involved in the nuclear emergency or radiation accident, a new type of X-band EPR resonant cavity for in vivo fingernail EPR dosimetry was designed and a homemade EPR spectrometer for in vivo fingernail detection was constructed. The microwave resonant mode of the cavity was rectangular TE101, and there was a narrow aperture for fingernail detection opened on the cavity’s wall at the position of high detection sensitivity. The DPPH dot sample and the fingernail samples were measured based on the in vivo fingernail EPR spectrometer. The measurements of the DPPH dot sample verified the preliminary functional applicable of the EPR spectrometer and illustrated the microwave power and modulation response features. The fingernails after irradiation by gamma-ray were measured and the radiation-induced signal was acquired. The results indicated that the cavity and the in vivo EPR dosimeter instrument was able to detect the radiation-induced signal in irradiated fingernail, and preliminarily verified the basic function of the instrument and its potential for emergency dose estimate after a radiation accident.

## Introduction

The triage and medical treatments for individuals involved in radiation accidents strongly depend on the ionizing dose^1,2^. Ionizing radiation generates free radicals in biologic materials, and most of them react and disappear immediately. While the radiation induced free radicals in water-deficient materials such as fingernail, tooth or bone are stable, and the quantity is proportional to the radiation dose^3,4^. The fingernail is a kind of material which is much easier to be acquired than tooth enamel or bone in most scenes. EPR spectroscopy is a specific sensitive method for the detection of free radicals. Therefore EPR spectroscopy of fingernail is a potential method for retrospective dosimetry after nuclear medical emergencies^5–8^.

The common method of EPR fingernail dosimetry is ex-vivo measurement of the fingernails. During ex-vivo measurements, the fingernails have to be cut into fragments. The clipping process generates the so-called mechanically-induced signal (MIS) which can seriously interfere with the radiation-induce signal(RIS), thus affecting the accuracy of dose evaluation. Therefore, the separation and elimination of the MIS caused by fingernail clipping is usually an important issue in the study of EPR fingernail dosimetry. The elimination of the MIS is an important effort to reduce the dose detection limit, improve the dose estimation accuracy, and would improve the practicability of the fingernail EPR dosimetry method for the diagnosis of the acute radiation syndrome (ARS)^9–11^.

A method of in vivo fingernail EPR dosimetry is proposed to reduce the negative effect of the MIS by avoiding the cutting process of the fingernail, and this method could improve the accuracy and feasibility of EPR dosimetry of fingernail^12,13^.

A commercial EPR spectrometer can be only used for ex-vivo measurement of the fingernail. There are several technical issues in the realization of in vivo EPR fingernail spectroscopy, and one of the most vital challenges is that the microwave resonator of the EPR spectrometer is not suitable for in vivo fingernail measurement. It is principally hindered by the closed structure of the resonant cavity and the additional problem of non-resonant absorption of microwave in the presence of lossy tissues.

Therefore, the fingernail has to be cut into pieces and then inserted into the sample tube. So the design of resonator for in vivo fingernail EPR measurement should be started before the method can become practical.

Ishii et al. proposed an X-band aperture cavity for in-vivo tooth dosimetry in early time^14,15^. Swartz H.M. team designed the L-band surface coil resonator for in vivo EPR tooth dosimetry[16]and made important progress in the L-band in vivo tooth EPR dosimetry^17–19^. And their team also designed several resonators for fingernail in vivo EPR spectroscopy^20,21^. In previous work, we also designed several kinds of aperture cavities for in-vivo tooth spectroscopy^22–27^. Based on these works, we newly designed an aperture X-band cavity which is special for in vivo fingernail EPR spectroscopy, and based on which, preliminary established an elementary prototype machine of in vivo fingernail EPR dosimeter. This paper detailed introduces the design of the cavity, and briefly introduced the other important units such as the magnet and the magnet modulation, and final construction of the EPR spectrometer. Some typical experiments of the DPPH dot sample and fingernail sample measurements were performed to preliminary verify the basic function of the instrument and its potential for radiation induced signal detection from the irradiated fingernails.

## Results

### The cavity and EPR spectrometer for in vivo fingernail dosimetry

#### The system design

**Figure 1:**
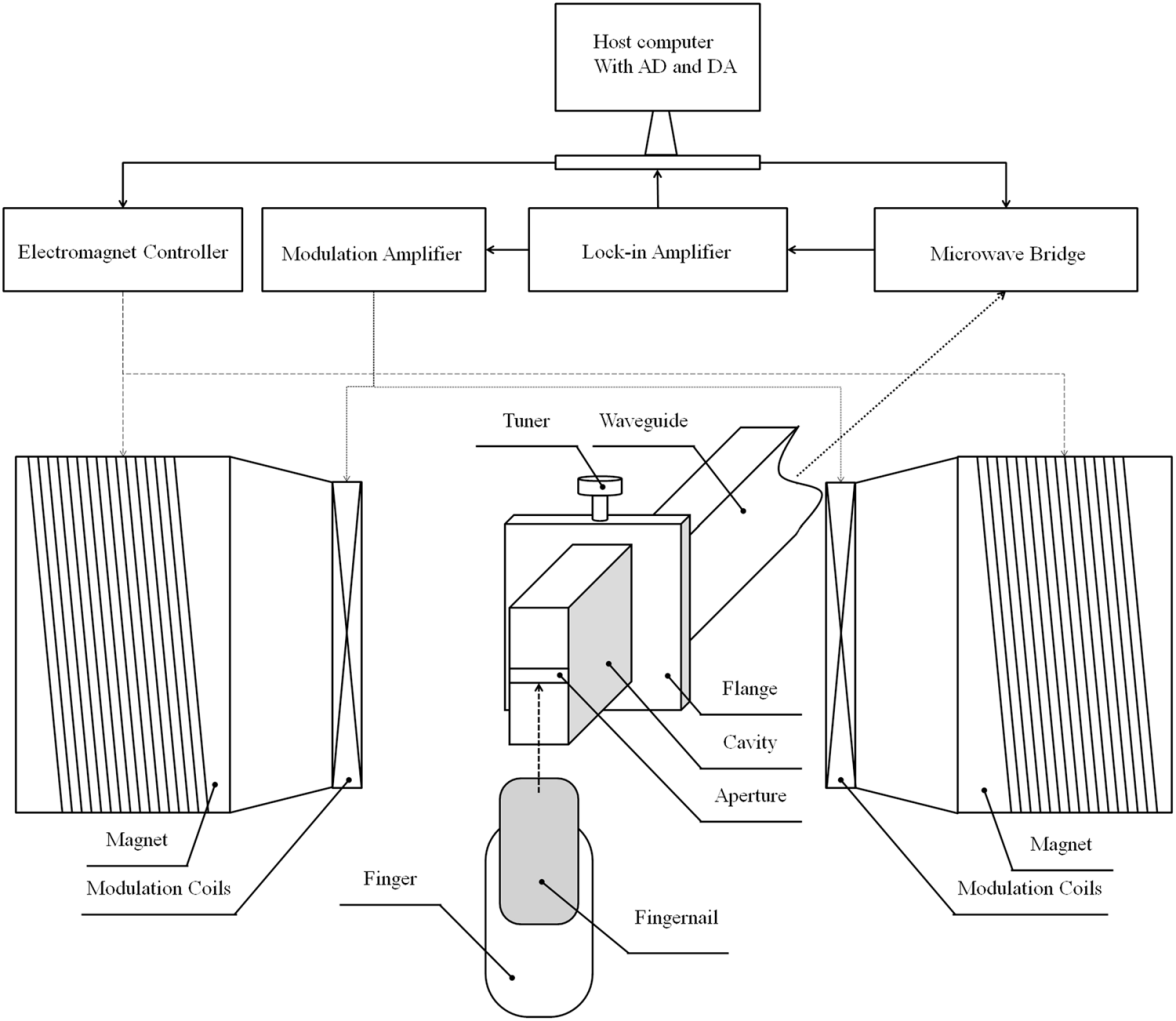
The system design of in vivo fingernail spectrometer.

According to the principal rules of EPR spectroscopy, the magnetic field, modulation, and microwave electromagnetic field should be applied to the sample in a certain direction, amplitude, frequency, and other parameters at the same time. We designed an in vivo EPR spectroscopy system as illustrated by Fig. 1. The main components of the spectrometer included a combined magnet unit with an electromagnet and permanent magnet, magnet modulation coils and its amplifier, microwave resonant cavity, microwave bridge, Lock-in amplifier, host computer installed with control and data process software, and DA-AD interface.

#### The cavity design

**Figure 2:**
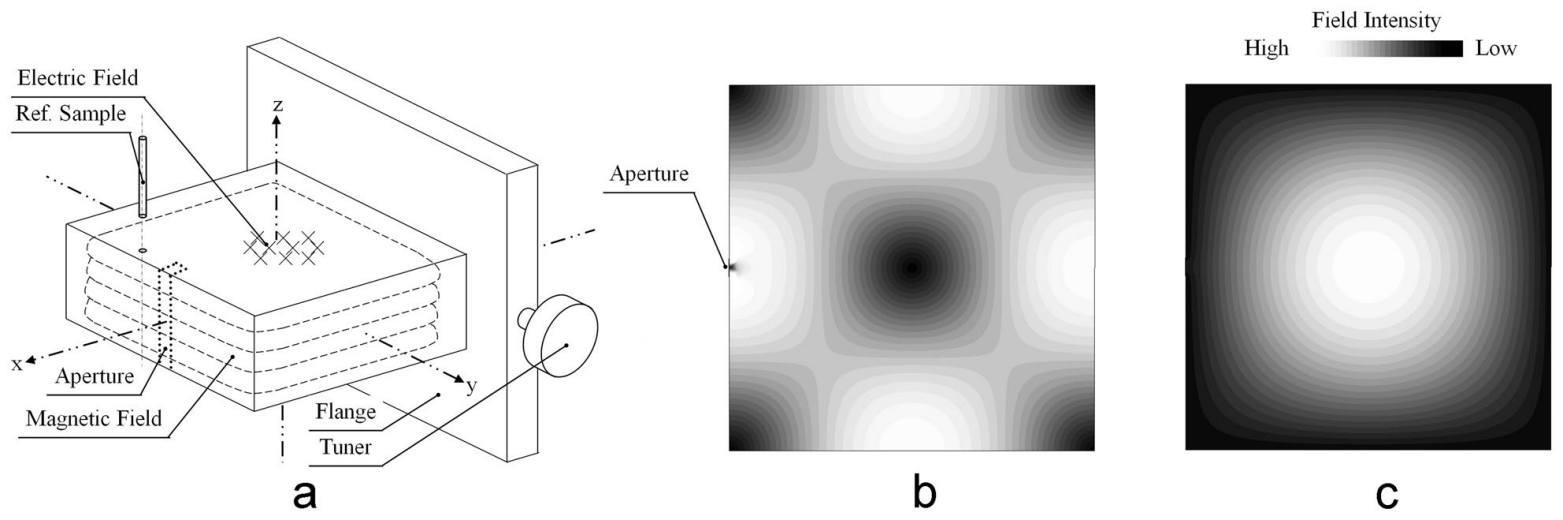
The cavity design. (**a**) The sketch of the aperture cavity for in-vivo EPR fingernail dosimetry. (**b**) Magnetic field distribution in the XOY plane. (**c**) Electric field distribution in the XOY plane.

Figure 2a shows the design sketch of the cavity. It was composed of the cavity body, detection aperture, flange and tuner, and the reference sample (ref. sample). The cavity body was based on a rectangular TE101 mode cavity. A rectangular detection aperture was opened on the cavity wall. The fingernail could be inserted into the aperture. The aperture was transfixed through the cavity body, in this way the magnet field and its modulation can be applied into the detecting aperture. Figure 2b,c showed the magnetic and electric field distribution in the cavity. It was consistent with the designed electromagnetic field configuration (The electric and magnetic field in Fig. 2a) of the TE101 mode resonant cavity.

The cavity design was confirmed by the finite element simulation software HFSS (High Frequency Structure Simulator). The simulation method and parameters were: Material, silver; Filling, dielectric air; Mode, Eigen mode; Cavity inner size, 22.1 mm × 7.0 mm × 22.1 mm; Aperture size, 9.0 mm × 1.1 mm. The simulation results were (1) the lowest microwave resonant mode was TE101, (2) the resonant frequency was 9.59 GHz. The resonant frequency and quality factor Q of the cavity were physically tested using the reflection method by a network analyzer. Some of the parameters and results were listed in Table 1. The design parameters, the simulation results, and the physical test results were mutually consistent with each other. The distribution of the microwave magnetic fields in the cavity was plotted by Fig. 2b. The aperture was located in the area of high magnetic field and thus sufficient microwave power could be achieved for the Zeenam levels splitting. The distribution of the microwave electrical fields in the cavity was plotted by Fig. 2c. The aperture was located in the area of low electrical field and the aperture was relatively narrow for microwave leaking, therefore, the non-resonant loss of microwave due to the lossy tissue could be avoided.

**Table 1:** Some tested parameters of the aperture cavity.

Items	Designed	Simulated	Tested
Length(mm)	22.10	22.10	22.10 ± 0.05
Width(mm)	7.00	7.00	7.00 ± 0.05
Height(mm)	22.10	22.10	21.10 ± 0.05
Aperture width(mm)	1.10	1.10	1.10 ± 0.05
Q value	–	–	3850
Frequency(GHz)	9.60	9.59	9.53

#### The integration of the spectrometer

**Figure 3:**
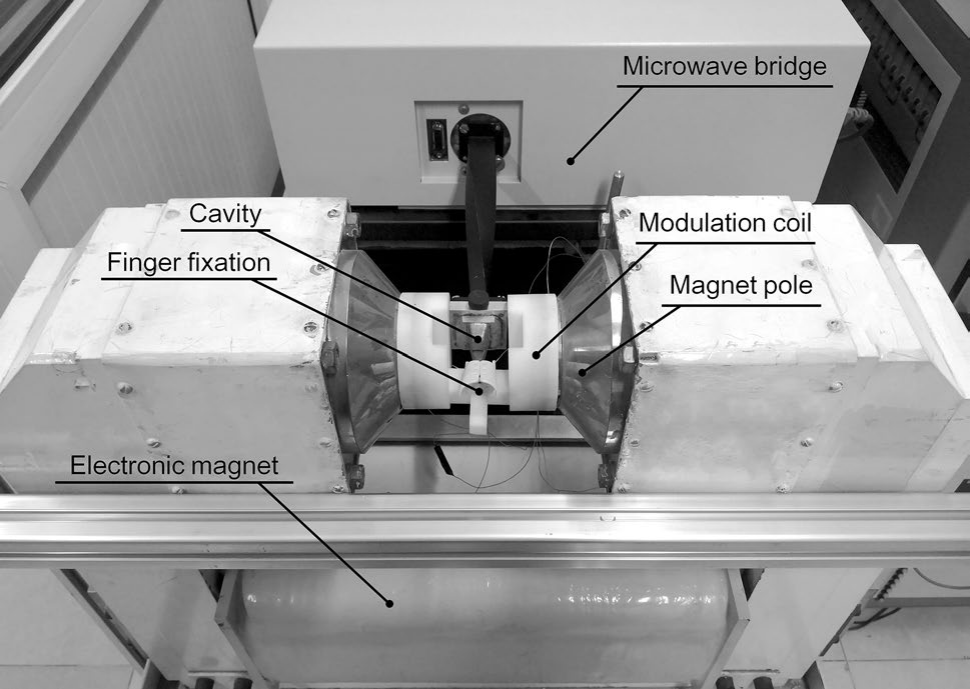
The X-band in vivo EPR spectrometer of fingernail dosimetry.

The final instrument constructed in our laboratory is shown by Fig. 3.

### EPR spectroscopy of DPPH dot sample

**Figure 4:**
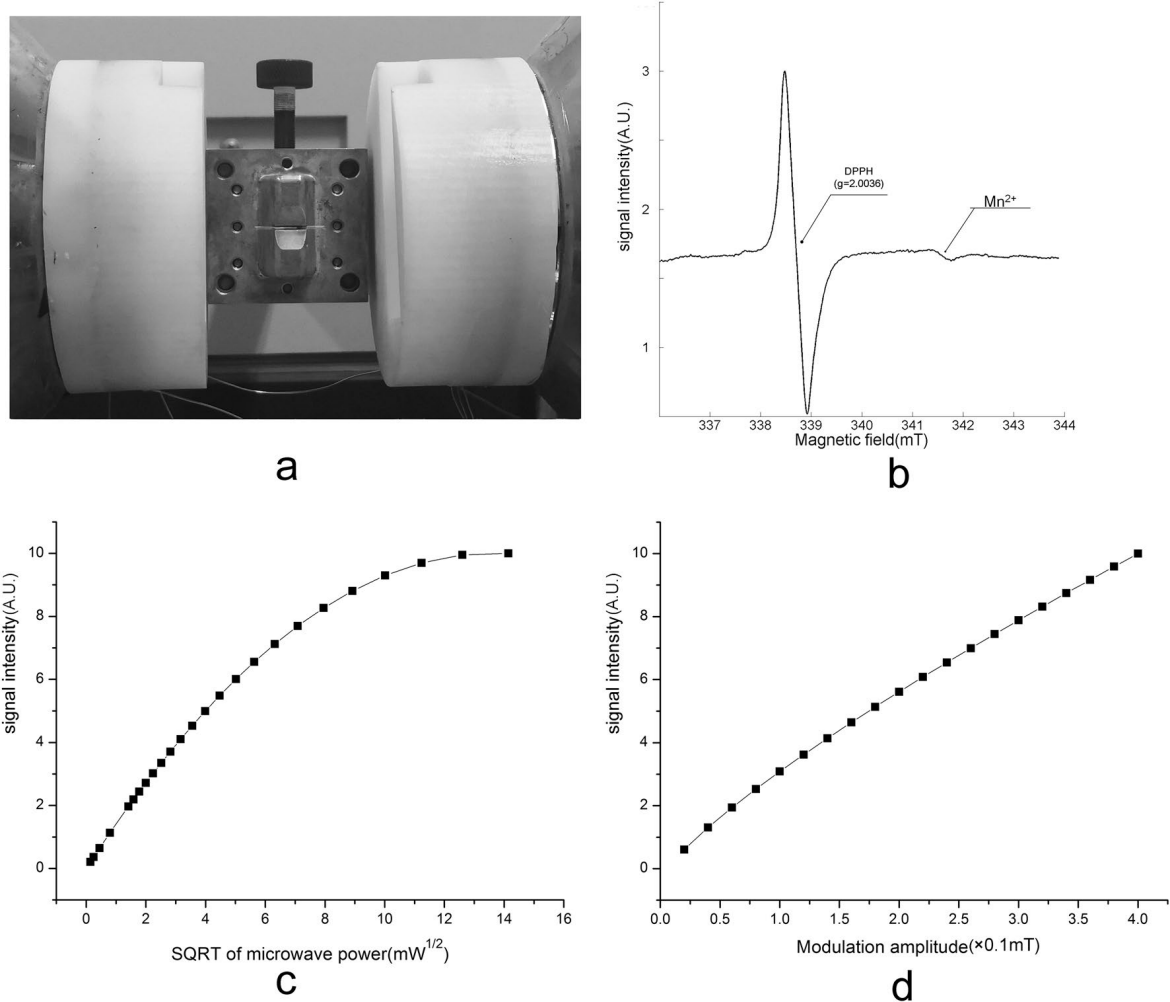
Result of EPR spectroscopy of a DPPH dot sample. (**a**) The DPPH sample inserted into the detection aperture of the cavity in the measuring status. (**b**) A typical EPR spectrum of the DPPH sample. (**c**) The relative signal intensity of the EPR spectra against the square root of power. (**d**) The relative signal intensity of the EPR spectra against the modulation amplitude.

Figure 4 shows the experimental results of the EPR spectroscopy of DPPH dot sample. Figure 4a shows the DPPH sample inserted into the detection aperture of the cavity in the measuring status. Figure 4b shows a typical EPR spectrum of the DPPH. Figure 4c,d shows the relative signal intensity of the DPPH spectra against the square root of microwave power and the magnet modulation amplitude. The signal intensity increased as the microwave power increased linearly with the sqrt of power up to 100mW, and then a lower rate of signal intensity indicated microwave power saturation. The signal intensity continuously increased proportionately as the modulation amplitude increased. The peak-to-peak EPR line-width of DPPH dot sample was significantly broadened when the modulation amplitude exceeded the intrinsic line width. These experimental results confirm that the newly assembled EPR spectroscopy system could function as designed, and that sufficient microwave power and modulated magnetic field could be applied into the detection aperture.

### The fingernail EPR spectroscopy

**Figure 5:**
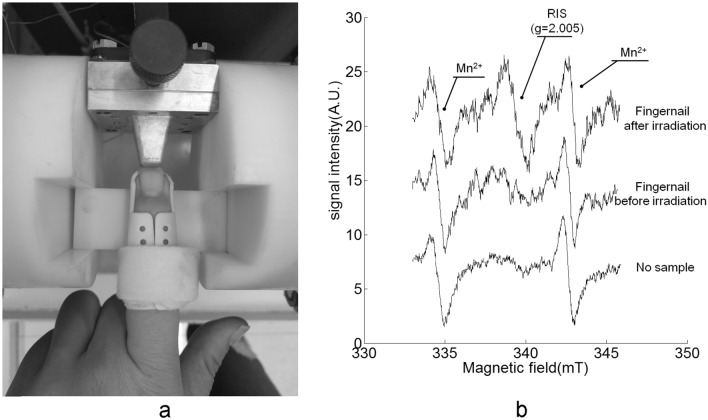
The in vivo EPR spectroscopy of fingernail. (**a**) The fingernail was inserted into the detection aperture in the state of being measured. (**b**) The EPR spectra of the in vivo measurement of the fingernails before irradiation and after irradiation by 6 Gy.

Figure 5a demonstrats the fingernail inserted into the detection aperture with only the cusp of the fingernail being detected. Figure 5b shows a typical fingernail EPR spectrum measured in vivo using a sample of an irradiated fingernail placed on top of the natural fingernail. Comparing the EPR spectrum of the fingernail before irradiation with the EPR spectrum of empty cavity (without fingernail sample), a background signal (BKS) could be found, which suggested the detection sensitivity of the spectrometer was in the ranger needed for fingernail EPR detection. The BKS intensity was approximately equivalent to the RIS intensity of 2–3 Gy. Comparing the spectrum of the fingernail before irradiation with the fingernail after irradiation of 6 Gy, a radiation-induced signal (RIS) could be found, which suggested the detection sensitivity of the spectrometer was available for dose assessment. Several radiation-induced signals have been found in the fingernail measurements^5–10^. The peak to peak value of RIS2(g = 2.005) was used as the signal amplitude of radiation-induced signal in this work, which was usually used in the previous studies on the feasibility of EPR dosimetry with nails^28–30^. The dose–response of this signal was found to be linear up to 300 Gy^6^.

## Discussion

The cavity is one of the most vital components for the in vivo EPR fingernail spectrometer system. The development of this X-band EPR cavity uses a narrow detection aperture opened on the cavity wall for in vivo fingernail detection, using TE101 rectangular mode resonator. The electromagnetic field distribution through an aperture in the TE101 mode rectangular cavity provides the possibility of external detection instead of detection inside the cavity. In the presence of an aperture the microwave magnetic field loops surround the cavity’s surrounding metal walls, and the intensity reaches the highest near the middle of the cavity wall. The detection aperture causes very little microwave electric field attenuation. The experiment with the DPPH dot sample measurement showed that the system could achieve satisfactory EPR spectroscopy. And the fingernail measurement showed that a combination of a background signal and a radiation-induced signal could be obtained, although at his time we do not have a practical means to resolve the RIS component.

The most significant feature of in vivo EPR fingernail dosimetry is that it eliminates the influence of the MIS signal caused directly by fingernail clipping in the dose evaluation. Compared with the study of in vivo tooth dosimetry^17,22,28^, the dose assessment precision and limit of the fingernail dosimetry might not be as good as tooth dosimetry (the standard error of prediction was approximately evaluated at 1.5 Gy for L-band spectroscopy of molar teeth)^17,18,28^. However, in vivo fingernail EPR dosimetry has some advantages in the potential for providing information and multiple sites (hands and feet) and future instrumental developments could achieve very competitive convenience for positioning, and speed of measurement^31^.

This work focused on the instrumental development, and the elimination of the MIS caused by fingernail clipping for the fingernail EPR dosimetry. The experiments in this paper were aimed to preliminary verify the designed function and demonstrate the feasibility of this method. However, there are still some more issues to be settled before this method becomes more available. The comprehensive dosimetric characteristics of in vivo fingernail EPR measurement still remain a series of further studies in the future work. The strong sensitivity of the radiation-induced signals in fingernails to humidity, especially to the direct contact with water, could result in the large deviation between nominal and EPR doses^29–32^. This may lead to inapplicability of EPR fingernail dosimetry in some circumstances. Other more studies and efforts should include the “real” irradiation experiment with UV to generate free radicals in fingernail; the normalization and calibration method of the signal intensity according to the size and the thickness of the fingernail; the improvement of the cavity of the spectrometer and so on.

## Conclusions

For the purpose of elimination the influence of the mechanically-induced signal (MIS) caused by fingernail clipping in the fingernail EPR dosimetry, the paper designed the in vivo spectrometer of fingernail with newly designed aperture cavity. The experiments with DPPH sample and fingernails were carried out to verify the design. The results indicated that the cavity and the in vivo fingernail EPR dosimeter was able to detect the radiation-induced signal in irradiated fingernail and could eliminate the MIS caused by fingernail clipping in the dose reconstruction of fingernail EPR dosimetry. This work was intended to promote the study of the EPR fingernail dosimetry and improve the practicability of this method in the nuclear emergency rescue.

## Methods

### The cavity design

#### The microwave resonant mode and electromagnetic field configuration

The microwave resonant mode inside the cavity was rectangular TE101, a resonant mode which was not appropriate in common ex-vivo EPR spectroscopy but had the superior features for in vivo EPR spectroscopy due to its electromagnetic field configuration inside the cavity. The electromagnetic field configuration in the TE101 mode cavity was illustrated in Fig. 2a. Most of the magnetic field component was distributed near the cavity surrounding walls. The electric field component was concentrated in the middle area of the cavity along the Z-axis. Figure 2b and Fig. 2c showed electromagnetic field distribution in the XOZ plane (Z = 0, the coordinate system was defined as Fig. 2a). TE101 mode was the dominant TE mode in the rectangular cavity and had the lowest cutoff frequency. Therefore, its electromagnetic field configuration was stable in the circumstances of interfering by resonant frequency or sample volume change. Compared with the TE111 and TM010 mode cavities we designed before^23,24^, the magnetic field component of the TE101 mode was more concentrated in the detection aperture area and thus lead to higher detection sensitivity than before.

#### The detection aperture

The aperture’s position was plotted by the dot line in Fig. 2a. It was positioned at the middle area of the cavity wall. The choice was made for: (1) The microwave magnetic field applied to the sample through the aperture was perpendicular with the external magnetic field and the magnetic modulation; (2)The aperture was located at the position of maximum microwave magnetic field component (H_1_) and (3) weak microwave electric field component (E_1_); (4)The aperture’s longer edge was parallel with the current flow on the surface cavity wall. The first requirement arose from the nature of the electron paramagnetic resonance condition. The second requirement was because the amount of microwave energy absorbed by the sample was proportional to H_1_^2^ below saturation; the third and fourth requirement minimized the microwave energy loss, which could lead to a deleterious effect on the detection sensitivity.

The detection aperture was rectangular. It was transfixed through the cavity body (along the z-axis) for the magnetic field and its modulation application. The aperture width was 1.1 mm (along the y-axis), which was suitable to set fingernails in and not too wide to cause microwave energy loss. The depth was 2 mm into the cavity (along the x-axis).

#### The dimension

The microwave resonant mode, resonant frequency, and resonant quality factor Q were dependent on the cavity’s dimension and material. The following conditions were referred to: (1) the X-band microwave system worked at about 9.60 GHz; (2) the resonant mode was TE101; 3) higher quality factor Q. The cavity was manufactured using copper material and plated with silver to reduce its surface resistance. Let the ideal rectangular resonant cavity length be *a,* width be b, and height be *d*. The solutions were given by

$$\left\{ \begin{gathered} a = 22.10\,{\text{mm}} \hfill \\ b = 7.00\,{\text{mm}} \hfill \\ d = 22.10\,{\text{mm}} \hfill \\ \end{gathered} \right.$$

#### The internal reference sample

There was an internal reference sample fixed near the detection aperture (ref. sample in Fig. 2a) to indicate the status of the in vivo EPR spectrometer and to calibrate the signal intensity. The internal reference sample containing Mn^2+^ in CaO encapsulated in a thin quartz capillary. The EPR signal of the reference sample could be measured synchronously with the EPR signal of the fingernail. The lines of Mn^2+^ and RIS of the fingernail could be included in a single spectrum and the two signals would be sufficiently separated.

#### The tune and coupling

The cavity was coupled to the microwave bridge through a circle coupling hole in the flange. The tuner was a screw bolt made of Teflon with a metal tip on its top, and it was fixed in front of the coupling hole. The coupling screw can be gradually turned to the hole to adjust the coupling coefficient between the cavity and the microwave system.

### The other units and the integration of the EPR spectrometer

#### The magnet

The magnet unit had a wide magnet poles gap. It was composed of a pair of permanent magnets and a set of electromagnetassembled on a C-shape yoke iron^33^. The main magnet field (about 335mT) was provided by alloy permanent magnet material of Nd-Fe-B. The scan of the magnetic field (about 0-10mT) was provided by the electromagnet. The scan of the electromagnet was driven by a set of programmable current source controlled through the host computer.

#### The magnetic field modulation

A special modulation device was developed for the modulation in the wide magnet gap. The modulation device included: (1) Modulation amplifier based on MOSFET Bridge that could supply high current than commercial equipment to drive the excitation coils. (2) A pair of excitation coils with the feed-back loops inside. The excitation coils generated magnet modulation in the magnet poles’ gap. The feed-back coils were used to indicate modulation amplitude by the induced currents that were proportional to the modulation amplitude. (3) Fixing frame to support the coils. The frame could be adjusted to control the coils in the particular angle and position between the poles. The modulation device could provide the modulated field higher than 1mT in the aperture area.

#### The commercial units

The microwave system was composed mainly of a commercial ready-made microwave bridge (from Bruker A300 EPR spectrometer) with a homemade water cooling device to secure the microwave generator. The signal receiver was also a commercial product of phase-sensitive lock-in amplifier (SR830). Some other units contained several power supply apparatus (Beijing Dahua) serving the microwave bridge, magnet, modulation amplifier, and others.

#### The host computer

The host computer was a notebook PC with a USB interface of the AD/AD board. The computer ran the software developed based on the Labview platform. The software function included the magnet scan control, data acquisition and storage, and the spectrum display.

### Measurement of DPPH dot sample

The experiment of DPPH dot sample measurement was intended to confirm if the newly assembled EPR spectroscopy system could realize the designed function, and to check the cavity design by verifying if sufficient microwave power and modulated magnetic field could be applied into the detection aperture. The DPPH(1,1-Diphenyl-2-picrylhydrazyl radical 2,2-Diphenyl-1-(2,4,6-trinitrophenyl)hydrazyl) powder was inserted in quartz capillary to form a sample of 1 mm in length and 1 mm in diameter and was used as a standard dot free radical sample. The DPPH dot sample was fixed in the center of the cavity aperture. Typical EPR spectroscopy’s parameters were: scan time 20 s, time constant 0.03 s, center field 340 mT, scan magnetic field 0 ~ 10 mT, microwave power 0.01 ~ 200 mW, microwave power 1 mW, and modulation amplitude 0.02 ~ 0.4 mT for modulation feature.

### Measurement of fingernail

The measurement of fingernail was used to preliminary verify the system design and the applicability of the cavity for in vivo fingernail EPR measurement. Because the fingernail could not be irradiated directly, a simulation method was performed. The first step, the fingernail was entirely clipped along the free margin of the fingernail and the free edge of the fingernail was clipped (the cracks or scratches should be avoided during the process), and then attached to the fingertip again to simulate the in vivo conditions; the second step, the background signal (BKS) was recorded through in vivo EPR measurements; the third step, the fingernail was irradiated by ^60^Co radiation source with dose of 6 Gy, then attached to the fingertip, and the radiation-induced signal (RIS) was recorded through in vivo EPR measurements. The detection aperture of the cavity was 2 mm in the depth direction, so the length of the fingernail (the length of the free edge) was at least 2.5 mm for in vivo EPR measurement. In this experiment, the fingernail length was longer than 4 mm to ensure only the cusp of the fingernail was inserted into the aperture and the cutting edge was left outside of the aperture, in which way, the MIS signal caused by fingernail clipping could be avoided.

The experiments were approved by the Medical Ethical Committee of Beijing Institute of Radiation Medicine (BIRM-MEC-20190115), and all the experiments were performed in accordance with relevant named guidelines and regulations. The informed consent was obtained from all participants.

The storage and laboratory environment conditions were: room temperate 20 °C; humidity 55%; container, airtight glass container. Typical ERP measurement parameters were: scan time, 5 s per scan, and 30 scans each measurement; magnet scan, 10 mT; microwave power, 5 mW; time constant, 0.03 s, center field 340 mT, modulation amplitude, 0.2 mT.
